# A tool for estimating antiretroviral medication coverage for HIV-infected women during pregnancy (PMTCT-ACT)

**DOI:** 10.1186/s41256-019-0121-3

**Published:** 2019-10-15

**Authors:** Bruce A. Larson, Nafisa Halim, Isaac Tsikhutsu, Margaret Bii, Peter Coakley, Peter C. Rockers

**Affiliations:** 1 Department of Global Health, Boston University School of Public Health, 801 Massachusetts Avenue, Boston, MA 02118 USA; 2 Kenya Medical Research Institute, U.S. Army Medical Research Directorate, Africa, Kenya; 3 U.S. Military HIV Research Program, Walter Reed Army Institute of Research, Silver Spring, MD USA; 4 Henry Jackson Foundation MRI, Kericho, Kenya

**Keywords:** HIV, Pregnancy, Prevention of mother-to-child transmission, Cascade of care, Pill count, Coverage, Proportion of days covered

## Abstract

**Background:**

In the typical prevention of mother to child transmission (PMTCT) of HIV cascade of care discussion or analysis, the period of analysis begins at the first visit for antenatal care (ANC) for that pregnancy. This starting point is problematic for two reasons: (1) a large number of HIV-infected women are already on life-long antiretroviral therapy (ART) when presenting for ANC; and (2) women present to ANC at different gestational ages. The PMTCT ART Coverage Tool (PMTCT-ACT), which estimates the proportion of days covered (PDC) with ART, was developed to address each of these problems.

**Methods:**

PDC is a preferred method to measure adherence to chronic medications, such as ART. For evaluating the PMTCT cascade of care, as indicated by PDC with ART over various time periods, a “starting point” based on a specific day before delivery must be defined that applies to all women (treatment experienced or naïve at the first ANC visit at any gestational age). Using the example of 168 days prior to delivery (24 weeks), PMTCT-ACT measures PDC with ART during that period. PMTCT-ACT is provided as a STATA do-file. Using an example dataset for two women (ID1 is treatment experienced; ID2 is treatment naïve), the details of each major portion of the tool (Parts 1–5) are presented. PMTCT-ACT along with the intermediate datasets created during the analysis are provided as supplemental files.

**Conclusions:**

Evaluating the PMTCT cascade of care requires a standard definition of the follow-up period during pregnancy that applies to all HIV-infected pregnant women and a standard measure of adherence. PMTCT-ACT is a new tool that fits this purpose. PMTCT-ACT can also be easily adjusted to evaluate other ante- and post-natal periods (e.g., final 4 weeks, final 8 weeks, complete pregnancy period, initial 24 weeks postpartum, time periods consistent with infant HIV testing guidelines).

## Background

﻿Ending the AIDS epidemic is goal 3.3 in the 2030 Agenda for Sustainable Development [1]. Prevention of mother-to-children transmission (PTMCT) remains an important component of the global epidemic for several reasons. First, the World Health Organization estimates globally that 1.3 million women with HIV were pregnant in 2018, and that nearly all young children newly infected with HIV are infected through mother-to-child transmission (MTCT) [2]. Second, the majority of female sex workers worldwide are mothers [3], and female sex workers are one of five key populations that affect the dynamics of HIV epidemics (both transmission to clients as well as their infants) [4]. And third, children-exposed to HIV through their mothers but uninfected (HIV-exposed but uninfected infants), are reported to have poorer growth outcomes than HIV-unexposed children [5].

Initiation of antiretroviral therapy (ART) during (or before) pregnancy, and adherence to ART throughout the antenatal and postnatal periods, significantly reduces MTCT [6]. In addition to reducing transmission through early ART initation and adherence, the severity of maternal HIV during pregnancy is reduced, which is then associated with lower mortality among HIV-exposed but uninfected infants (see, for example, [7]).

Mother-to-child transmission of HIV can occur during pregnancy, delivery, and the postpartum period [6]. However, adherence to ART during pregnancy remains poor in resource-poor settings, prompting systematic monitoring of treatment initiation and adherence across the prevention of mother-to-child transmission (PMTCT) of HIV cascade of care by treatment programs and researchers (see, e.g., [3] for a recent review). Knowledge about the timing and duration of poor adherence across the PMTCT cascade of care can be used to target support services or new interventions to support international efforts to eliminate MTCT by 2030 [8].

In the typical PMTCT cascade of care discussion or analysis, the period of analysis begins at the first visit for antenatal care (ANC) for that pregnancy (see, e.g., as outlined for example in [9–17]). This starting point is problematic for two reasons. First, a large proportion of HIV-infected women presenting for ANC are already on life-long ART; and (2) women present to ANC at different gestational ages. Late initiation of ART during pregnancy increases risks of MTCT [18], and a longer duration on ART at delivery reduces risks of transmission [19]. However, longer duration on ART without good adherence increases risks of viral failure, which in turn increase risks of transmission [20].

Evaluating the PMTCT cascade of care, specifically focused on ART initiation and adherence for pregnant and post-partum women in the era of “treatment for all”, first requires a standard definition of the follow-up period during pregnancy that applies to all women and standard measures of adherence. As part of an evaluation of PMTCT service delivery study in Kenya [21], we have reconceived a unified PMTCT cascade of care that includes treatment experienced and naïve HIV-infected women presenting for ANC at different gestational ages. And second, a recent review of the effectiveness of interventions to improve adherence in pregnant women on ART in sub-Saharan Africa concluded that most studies used unvalidated, self-reporting measures of adherence [22]. As an alternative or complementary approach, a standard measure for adherence to chronic medictions adherence, the proportion of days covered (PDC) [23–26], can be used to measure adherence for evaluating PMTCT service delivery and interventions to improve adherence.

The purpose here is to present the updated PMTCT cascade of care and provide an empirical tool (the PMTCT ART Coverage Tool) to estimate PDC with ART over any given window of time during pregnancy.

## Methods

### An updated PMTCT Cascade of care

**Fig. 1:**
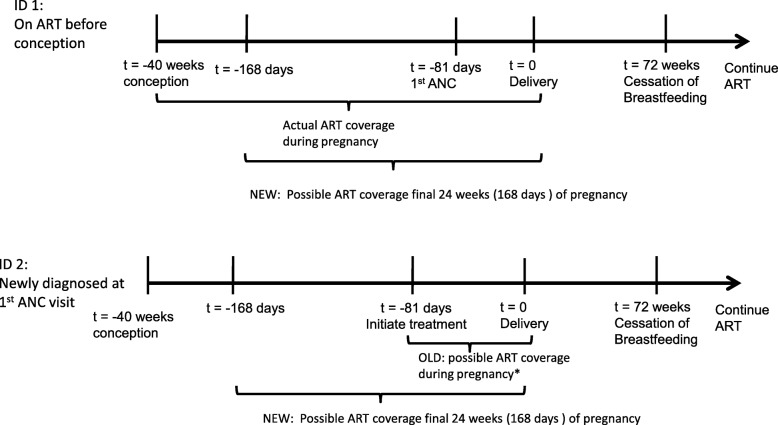
Reconceiving the PMTCT Cascade of Care for women in the antenatal period

Figure 1 summarizes an updated PMTCT cascade of care for two HIV-positive pregnant women presenting for their first ANC visit at the same stage of pregnancy – 81 days before delivery, which is approximately 28 weeks gestation. Patient ID1 represents the category of pregnant women who are treatment experienced when presenting for ANC, while Patient ID2 represents the category of pregnant women who are treatment-naïve when presenting for ANC.

As noted above, in the traditional PMTCT cascade of care, the period of analysis begins at the date of first ANC, which ignores two issues: (1) there is large variation in gestational age at the time of first ANC; and (2) treatment-experienced women have ART coverage prior to their first ANC visit. In Fig. 1, treatment-naïve ID2 has a maximum of 81 days during pregnancy covered by treatment. Treatment-experienced ID1, who initiated before conception, has a maximum of 280 days covered (assuming 40 weeks of pregnancy). While the example in Fig. 1 assumes both women present at the same gestational age, gestational age at the first ANC visit is largely ignored in the standard PMTCT cascade of care even through some women present early in their pregnancy (perhaps < 12 weeks gestation), while an important share present late (perhaps > 35 weeks).

To evaluate treatment-experienced and treatment-naïve patients equally, we propose an alternative to the traditional PMTCT cascade of care during the antenatal period. Rather than using the first ANC visit as the first relevant time period in the PMTCT cascade, our approach allows analysts to specify any window of time prior to delivery for evaluating ARV coverage during pregnancy for both treatment-naïve and treatment-experienced women. This window could be the complete pregnancy period or some pre-specified window such as the final 24 weeks of pregnancy. In the example in Figs. 1, 24 weeks (168 days) was chosen as an example. In addition, patients initiated on ART with relatively high CD4 cell counts have a high probability of being virally suppressed within 24 weeks [27], which is important because maternal viral load is a key factor in mother-to-child transmission [28].

To measure ART coverage during the pre-specified window, we apply the concept of proportion of days covered (PDC), which calculates the number of days covered by a prescription or dispensing records and divides by the number of days in the measurement period [23–25]. For the PMTCT cascade, measuring PDC—hereafter referred to simply as coverage—requires counting the number of days over a window of time (the final 24 weeks of pregnancy in Fig. 1) a patient has one or more days of ART on hand each day (e.g., number of pills for a once-a-day triple fixed-dose combination). The counting process needed to estimate coverage over a window of time during pregnancy is complicated because patients can return before their pills run out, or return exactly on time, or return after gaps in coverage.

To facilitate estimation of ART coverage during pregnancy, we developed the PMTCT ART Coverage Tool (PMTCT-ACT) that can be easily used to estimate coverage over any window during the pregnancy period. PMTCT-ACT can also be easily applied to estimate coverage for additional periods of time, for example that align, for example, with infant HIV testing at 6 weeks, 24 weeks, and 48 weeks postpartum.

### Using PMTCT-ACT

We provide an overview of the PMTCT-ACT analysis process with an example in this section. PMTCT-ACT is currently organized using STATA (works in versions 13+) as a 5-step do-file (see Additional file 1, PMTCT-ACT.do). PMTCT-ACT along with the files used and discussed in this section are provide as supplemental electronic files. The PMTCT-ACT.do file can also be opened in a text editor for analysts who do not have access to STATA and who would like to adapt the tool for use with other software packages (e.g., SAS, R). In addition, a pdf version of the tool (Additional file 2, PMTCT-ACT.pdf) is also provided for viewing.

### Prepare a basic dataset

To use PMTCT-ACT, a basic panel dataset needs to be developed that contains the five variables included in Table 1. The data in Table 1 is an example dataset for the two women (ID1 and ID2) described in Fig. 1. In Table 1, both women presented for ANC on February 10, 2017 (variable name *ANCdate*) and delivered on May 2, 2017 (*deliverydate*). While each woman presented for her first ANC visit 81 days prior to delivery (day − 81), ID1 was treatment-experienced at the time having initiated ARVs in June of 2015, while ID2 was treatment-naïve and initiated at the first ANC visit. Each row in the dataset shows the patient’s unique number (*ID*), a date when ARVs were prescribed/provided (*date*), and the days of ART prescribed (*daysarvs*). For example, for a “one-month” prescription provided as a once-a-day fixed combination (such as TDF + 3TC + EFV) in a 30-pill pack, *daysarvs* would be equal to 30.

**Table 1:** Example panel dataset for treatment-experienced and treatment-naïve patients

*ID*	*date*	*daysarvs*	*ANCdate*	*deliverydate*
1	18-Jun-15	15	10-Feb-17	2-May-17
1	12-Aug-15	60	10-Feb-17	2-May-17
1	9-Oct-15	90	10-Feb-17	2-May-17
1	17-Feb-16	60	10-Feb-17	2-May-17
1	19-Apr-16	90	10-Feb-17	2-May-17
1	29-Jul-16	90	10-Feb-17	2-May-17
1	21-Nov-16	100	10-Feb-17	2-May-17
1	10-Feb-17	30	10-Feb-17	2-May-17
1	12-Apr-17	60	10-Feb-17	2-May-17
1	19-Jun-17	30	10-Feb-17	2-May-17
1	25-Jul-17	60	10-Feb-17	2-May-17
1	29-Sep-17	60	10-Feb-17	2-May-17
2	10-Feb-17	15	10-Feb-17	2-May-17
2	25-Feb-17	30	10-Feb-17	2-May-17
2	27-Mar-17	30	10-Feb-17	2-May-17
2	26-Apr-17	60	10-Feb-17	2-May-17

The dataset in Table 1 is provided as Additional file 3, **0 – Basic dataset.dta,** as a STATA data set. For completeness, the initial dataset (**0 – Basic dataset.dta**) includes a third possible category of women (ID3) who initiate ART after delivery. While such women will have 0 coverage during pregnancy, they may/will have coverage during the post-natal period (if PMTCT-ACT is used, for example, to estimate coverage during the initial 24 weeks post-natal period to coincide with an infant HIV test also at 24 weeks).

How this basic dataset is developed depends on records systems at the site(s) included in the analysis. With electronic pharmacy records, it is relatively straight forward to obtain data on the date ARVs are provided and the quantity provided. With paper-based medical records, the effort required to extract and enter data is substantial, but the authors and colleagues have completed several ‘cost-outcomes’ studies that included the same type of information [29–34]. Identifying the date of the first ANC visit for that pregnancy, along with the delivery date (infant’s birth date) depends largely on the quality of these types of medical records, typically found in a maternal and child health (MCH) clinic. As long as the identification number used for the mother is the same in both the general HIV clinic (or outpatient clinic) where the mother received care and treatment prior to her pregnancy and the MCH clinic where she receives care in the antenatal and postnatal periods, creating such a dataset is not too problematic. In other situations, without good records, obtaining such data may itself be difficult.

As an additional note, it is likely the case that a woman could present for ANC at the clinic but deliver at an alternative location, such as at home or a hospital not linked to the MCH clinic. In such situations, it is possible that MCH medical records do not include a date of birth. Gestational age at the first ANC visit (typically included in patient records) can be used to estimate a date of delivery if needed.

### Run PMTCT-ACT

PMTCT-ACT is organized into 5 parts (or do file sections). Each is described briefly below and a summary table of the 5 steps is provided in Table 3 at the end of this section.

#### *PMTCT-ACT part 1*

Once the basic dataset is prepared as outlined in Table 1 and the dataset is open in STATA,

PMTCT-ACT Part 1 creates two new variables and then expands the dataset into a balanced panel dataset (equal number of observations for each ID). A new variable is created, *dateonart,* equal to the first date in the dataset for each ID (i.e., the date of ART initiation). A second new variable, *daysonarvs,* counts the number of days from *dateonart* for each value of *date*. Note that *daysonarvs* is now the time variable in the panel dataset (not date).

In Part 1, the analyst needs to consider the full follow-up time period that will be included in the analysis. There are two issues here: (1) some women might have initiated ART many years before the delivery date, so the *daysonarvs* variable in the panel dataset must be large enough to cover that period of time; and (2) additional time periods may need to be included to cover the full follow up period needed for the analysis. In the example dataset, ID1 initiated ART more than 2 years before delivery (834 days before delivery to be exact), so the expanded panel dataset created in Part 1 automatically has these 834 days for all IDs. To make sure the period covered by *daysonarvs* is long enough for the analysis (e.g., to cover the pregnancy periods), extra days are added for each ID. For the example provided here, an additional 200 days are added onto the dataset for each women (so *daysonarvs* begins at *j* = 0 (day of initiation) and continues through *j* = 1034). The dataset could be saved at this stage (see **1 -- Full Panel after Part 1.dta** provided as Additional file 4).

#### *PMTCT-ACT part 2*

PMTCT-ACT Part 2 ‘reshapes’ the panel dataset (many observations for each ID) to a ‘wide’ dataset with one observation for each ID. Because there are 1034 observations for each ID, this reshaping creates a dataset with a rather large number of variables. At the end of Part 2, the ‘wide’ dataset has 1035 variables, daysarvs0 – daysarvs1034, along with the 4 variables that did not change across time in the panel (*ID, date_delivery, date_ANC, date_beginart*).

The key variables for counting coverage are the *daysarvs#*, where # is the number of days the patients was on ART (*daysonart* variable created in Part 1). For example, each patient received 15 days of ARVs on *daysonart* = 0. As a result, in the ‘wide’ data set, the new variable *daysonarvs0* = 15 for each. Neither ID received additional ARVs on *daysonart* = 1, so *daysonarvs1* =. (missing value generated in the reshaping process). Only ID1 received ARVs on day 55 on ART (60 days of ARVs), so *daysarvs55* = 60 for ID1 but 0 for ID2, and so on.

The wide dataset could be saved at this stage (see **2 – Wide dataset after Part 2.dta** provided as Additional file 5).

#### *PMTCT-ACT part 3*

PMTCT-ACT Part 3 is a “pill-count calculator” that counts for each day in the dataset the number of days of ART “on hand” for that patient. For example, if a woman received 15 days of ART on the day she initiates treatment, then on day 0 she has ART for 15 days. On the next day, day 1 on ART, she has pills for 14 days. If a patient received a drug refill exactly on the day she ran out of ART or later after she ran out of ART, counting pills available each day would be simple. However, it is possible and not uncommon for patients at times to return ‘early’ and obtained additional ARVs. For analysts who may wish to adapt PMTCT-ACT to SAS or other software, this portion of PMTCT-ACT is similar to pill count calculators developed previously in SAS [23] and STATA [35].

At the end of Part 3, the dataset is reshaped again into a panel dataset with *daysonarvs* as the time variable. The dataset now has six variables, with the variable *drugs_* showing the number of days of ART each ID has at the beginning of each day for all days in the dataset.

This new panel dataset can be saved at this stage (see **3 -- Final panel dataset with pill counts after Part 3.dta** provided as Additional file 6).

#### *PMTCT-ACT part 4*

At this stage, the panel dataset uses *daysonarvs* as the time variable, with *daysonarvs* = 0 representing the day of ART initiation. Part 4 of PMTCT-ACT creates a new time variable, *daystodelivery*, that shows the date relative to the date of delivery. This new time variable is needed to assess if the patient could have ART at the beginning of each day relative to delivery. In addition, a simple variable, *hasarvs*, is created that equals 1 if the patient has ARVs at the beginning of the day, or 0 if not, based on the results from Part 3. The final section of Part 4 reorganizes the data set into a balanced panel dataset with *daystodelivery* as the time variable, with *daystodelivery* beginning at − 280 (an estimate of conception based on 40 weeks gestation) and + 365 to cover the first year after delivery. The numbers can be easily adjusted to allow a longer window of time before and after delivery.

This final panel dataset could be saved at this stage (see **4 – Final Panel dataset after Part 4** provided as Additional file 7).

#### *PMTCT-ACT part 5*

The final panel dataset (**4 – Final Panel dataset after Part 4)** contains all the information (variables) needed to count how many days over a certain period of time the patient was covered with ART. For the example presented here, we will focus on the final 24 weeks of pregnancy (from *daystodelivery* = − 168 to *daystodelivery* = 0).

In Part 5, for this example, the dataset is ‘collapsed’ over the final 24 weeks of pregnancy into a new dataset with one observation per ID with the following variables:

- *daysonarvs* = the number of days from treatment initiation to delivery;
- *date_delivery* = the date of delivery;
- *date_ANC* = the date the patient presented for antenatal care;
- *date_beginart* = the date the patient initiated ART; and
- *hasarvs* = the number days covered with ART over the collapsing period (potential days covered).

A new variable is then created, *coverage_24*, which is the proportion of days over the period covered with ARVs (24 weeks = 168 days). An additional 0/1 coverage variable is created, *coverage_85p*, which equals 1 if coverage is at least 85% during the collapsing period. This coverage variable can be easily adjusted for other levels. The final collapsed dataset is then saved as **5 – Final outcome 24 weeks to delivery.dta** (provided as Additional file 8).

Note that PMTCT-ACT Part 5 can be easily adjusted to measure ART coverage over other time periods relevant for the PMTCT cascade of care, such as the first 6 and 24 weeks post-partum, which coincides with typical infant HIV testing guidelines. In addition, because the dataset after Part 4 contains the time variable *daysonarvs,* ART coverage for other types of patients (adults, adolescents) over various time periods after initiation can be easily analyzed.

The final dataset created by PMTCT-ACT using the data from Table 1 is provided in Table 2.

**Table 2:** Final dataset for ART coverage

ID	daysonarvs	date_delivery	date_ANC	date_beginart	hasarvs	coverage_24	coverage_85p
1	684	2-May-17	10-Feb-17	18-Jun-15	151	0.898810	1
2	81	2-May-17	10-Feb-17	10-Feb-17	82	0.488095	0

For ID1, ART coverage over the final 24 weeks of pregnancy (*coverage_24*) is 0.89 (89% of days covered), so *coverage_85p* = 1. For ID2, *coverage_24* is 0.48, and *coverage_85* = 0. Additional variables could then be merged into this dataset (e.g., ID demographics, intervention arm for trials) for further analysis.

Table 3 provides a brief summary of the PMTCT-ACT steps used to create Table 2.

**Table 3:** A summary of the PMTCT-ACT process

Part 1	• Create a new time variable (days on ART for each visit date relative to the date of ART initiation). • Expand the data set to a full, balanced panel that covers the time period needed for the analysis (e.g. from the earliest date of ART initiation through the latest date of delivery for all patients in the analysis).
Part 2	• Reshape the panel dataset (a “long” dataset) to a “wide” dataset.
Part 3	• Use the pill count calculator to count the number of days of ARVs on hand for each day in the wide dataset. • Reshape the dataset from wide to long (back to a panel dataset).
Part 4	• Creates a new time variable for each date that is the number of days from that date to the date of delivery. • For each date, create a new indicator variable (hasarvs) equal to 1 if the patient has 1 or more days on ART on hand that day (that day is potentially covered with ART).
Part 5	• Collapse the panel dataset over a specified period of time. • The example provided is the final 24 weeks of the pregnancy (e.g., days to delivery −168 to 0). • Create a final outcome measure (coverage with ART during the final 24 weeks of pregnancy).

## Discussion

The purpose of this paper was to update the standard presentation of the PMTCT cascade of care so that it can be applied consistently to pregnant women already on ART at their first visit for antenatal care as well as those newly diagnosed. Rather than beginning the cascade with the first ANC visit, a standard window of time prior to delivery is specified. In the example here, 24 weeks before delivery is used because rates of viral suppression are high after 24 weeks if patients adhere to ARV medications.

Once the starting point for the cascade is defined, the next question becomes the metrics to use to evaluate PMTCT service delivery and outcomes. The proportion of days (PDC) covered is a standard medication adherence measure that can be used consistently for women already on ART when presenting for antenatal care and women newly diagnosed with HIV at or near their first ANC visit. As noted in the introduction, the proportion of days covered has been used previously as a metric in a cohort study evaluating adherence in PMTCT programs [26] and in a currently on-going randomized evaluation of an intervention to improve PMTCT service delivery [21].

In Table 2, the pregnant woman who initiated ART at her first ANC visit (ID2) had 49% of the days covered with ARVs during the final 24 weeks of pregnancy, which is substantially less than the 90% of days covered for ID1 (already on ART at her first ANC visit). However, in the traditional cascade of care, which begins at the first ANC visit, ID2 would have almost perfect coverage of ARVs during her final 81 days of pregnancy, which obscures the fact she initiated ART fairly late in her pregnancy.

Future research remains needed to develop a consensus on a ‘standard’ window during delivery for measuring coverage. Given that high rates of viral suppression are achieved with consistent adherence to antiretroviral medications, viral suppression in the mother protects her health and minimizes risks of congenital HIV in her newborn [36], and high rates of viral suppression are achieved after 24 weeks on ART [27], our use of at least 24 weeks as an example for PMTCT-ACT is logical. However, PMTCT-ACT can be easily adjusted to any other window of time.

As noted in the introduction, adherence to ART during pregnancy remains poor in resource-poor settings, prompting systematic monitoring of treatment initiation and adherence across the prevention of mother-to-child transmission (PMTCT) of HIV cascade of care by treatment programs and researchers (see, e.g., [3] for a recent review). We expect that any researcher evaluating PMTCT programs, or interventions designed to improve implementation, could use this tool to measure adherence for primary study outcomes. Although the basic data required for the implementation of PMTCT-ACT could be extracted from paper-based medical records in clinics without electronic medical record systems (EMRS), the continued expansion of EMRS in resource-limited setting will further facilitate the application of PMTCT-ACT for evaluating PMTCT service delivery. In such systems, a key feature is to make sure the mother’s records are cleanly linked with the infant’s records so that, for example, date of birth of the infant can be easily identified to develop the basic dataset needed for this analysis (as shown in Table 1). For example, only 22% of mother-infant files could be linked in an evaluation of PMTCT services in Malawi [26].

## Conclusion

Evaluating the implementation of PMTCT services and guidelines requires a standard definition of the follow-up period during pregnancy for all women and a standard measure of adherence during pregnancy. The proportion of days covered with ART medications is one logically metric, and the tool provided with this manuscript (PMTCT-ACT) can be freely used to calculate coverage.

We welcome use of the tool by all who might find it valuable, though we expect that the tool will be most useful for researchers evaluating PMTCT programs or interventions designed to improve program implementation. In addition, national AIDS programs could also use the tool, at least in part, as part of their monitoring and evaluation activities. The authors welcome any comments, recommendations, or possible additions to the tool from users.

## Supplementary information

**Additional file 1.** PMTCT-ACT.do. Information on file format. STATA do file (version 15).

**Additional file 2.** PMTCT-ACT.pdf. Information on file format. Portable Document Formatted file (pdf).

**Additional file 3.** 0 – Basic dataset.dta. Information on file format. STATA dataset (version 14 and 15).

**Additional file 4.** 1 – Full Panel after Part 1.dta. Information on file format. STATA dataset (version 14 and 15).

**Additional file 5.** 2 – Wide dataset after Part 2.dta. Information on file format. STATA dataset (version 14 and 15).

**Additional file 6.** 3 – Final Panel dataset with pill counts after Part 3.dta. Information on file format. STATA dataset (version 14 and 15).

**Additional file 7.** 4 – Final Panel dataset after Part 4.dta. Information on file format. STATA dataset (version 14 and 15).

**Additional file 8.** 5 – Collapsed final outcome dataset after Part 5.dta. Information on file format. STATA dataset (version 14 and 15).

## Data Availability

All data and methods used are provided as supplemental files.
