# Supplementary material for: A tool for estimating antiretroviral medication coverage for HIV-infected women during pregnancy (PMTCT-ACT)
Source: Glob Health Res Policy. 2019 Oct 15;4:29. doi: 10.1186/s41256-019-0121-3 (PMC6794749; doi:10.1186/s41256-019-0121-3)
Supplement: Supplementary file 2 — Additional file 2. PMTCT-ACT.pdf. Information on file format. Portable Document Formatted file (pdf). [file 41256_2019_121_MOESM2_ESM.pdf]

```
1
2
3   ********************************************************************************************************************************
    ****************
4   *
5   *
6   *
7   *                                       The PMTCT ART Coverage Tool (PMTCT-ACT)
8   *                                       (Original version, January 18, 2019; Updated April 11, 2019)
9   *
10  *                                       Developed by Bruce Larson, Nafisa Halim, Peter Rockers
11  *
12  *
13  *                                       This tool is freely available for use and further adaptation or development.
14  *
15  *                                       Please reference as:  Larson, BA, Halim, N, Rockers, P, The PMTCT ART Coverage
    Tool (PMTCT-ACT),
16  *                                       Boston University School of Public Health, Boston, MA, USA, 2019
17  *
18  *                                       There are 5 main parts to PMTCT-ACT (this do file).  Each is labeled below.
19  *
20  *
21  *
22  *
23  ********************************************************************************************************************************
    ****************
24
25
26
27  ********************************************************************************************************************************
    ****************
28  *
29  *
30  *
31  *
```

```
35  *   Use 0 -- Basic dataset.dta
36  *
37  *
38  *
39  *   RUN  PART 1 OF THIS DO FILE TO CREATE DAYS ON ART FOR EACH DATE FOR EACH PATIENT IN THE DATA SET.
40  *
41  *
42  *
43  ********************************************************************************************************************************
    ******************
44
45
46
47  sort ID date
48
49  by ID: egen dateonart = min(date)                    /* first prescription date in dataset is the date on ART  */
50
51  by ID: generate daysonart = date - dateonart         /* For each date, how many days is the patient on ART */
52
53  format %td dateonart                                 /* Just formats dateonart variable */
54
55  drop date
56
57  move daysonart daysarvs
58
59
60  tsset ID daysonart                                   /* sets as panel data for STATA but unbalanced*/
61
62  tsfill, full                                         /* creates a balanced panel with daysonart going from 0 to 834
    (max in the data)*/
63
64  tsappend, add(200)                                   /* adds extra observations to have the time variable span the
    length of follow up needed */
65
66
```

```
would need to be expanded.

 *  Save dataset as 1 - Full Panel after Part 1.dta

*************************************************************************************************************************
*
*
*                                                    PART 2
*
*
* PART 2  -- CREATE WIDE DATASET (ONE OBSERVATION PER ID) TO PREPARE DATASET FOR THE PILL COUNT CALCULATOR
*
*
*
*
*
*************************************************************************************************************************

reshape wide deliverydate ANCdate dateonart daysarvs, i(ID) j(daysonart)

* THE MOVE/RENAME CODE BELOW CLEANS UP THE THREE TIME VARIABLES THAT ARE CONSTANT FOR EACH PATIENT.

move deliverydate0 daysarvs0

rename deliverydate0 date_delivery

move ANCdate0 daysarvs0

```

```
105
106 rename dateonart0 date_beginart
107
108 drop deliverydate* ANCdate* dateonart*
109
110
111 * Save dataset as 2 - Wide Data Set after Part 2.dta
112
113
114
115
116 ********************************************************************************************************************
    **********************
117 *
118 *
119 *                                                    PART 3
120 *
121 *
122 * PART 3  --    THIS PART OF THE DO FILE INCLUDES THE "PILL COUNT CALCULATOR"
123 *
124 *
125
126 * TO BEGIN, A VARIABLE "DAYS_WITHOUT" IS CREATED THAT HAS TO BE LARGE ENOUGH TO COVER THE MAXIMUM TIME PERIOD NEEDED FOR
    YOUR ANALYSIS.
127 *
128 * FOR EXAMPLE HERE, WE USE 1000 DAYS (BECAUSE ID 1 WAS ON ARVS SLIGHTLY MORE THAT 2 YEARS BEFORE DELIVERY, 1000 DAYS IS
    ADEQUATE)
129 *
130 *
131 *
132 * IF SOMEONE STARTED ARVS 5 YEARS BEFORE THEIR DELIVERY DATE, YOU NEED TO HAVE AT LEAST 5*365 HERE TO COVER THE PERIOD TO
    DELIVERY.
133 *
134 * IF A STUDY IS GOING TO FOLLOW WOMEN INTO THE POST PARTUM PERIOD (FORE EXAMPLE 18 MONTHS AFTER DELIVERY) THEN MORE DAYS
    NEED TO BE ADDED.
```

```
**********************


set more off

gen days_without = 1000                                          /* ONE */
gen drugs_0 = daysarvs0
replace days_without = 999 if drugs_0 != 0                       /* TWO 1000 - 1  */
gen drugs_has = .

local j = 0
forvalues i = 1(1)999 {                                          /* THREE 1000 - 1 */
    local j = `i' - 1
            gen drugs_`i' = drugs_`j' + daysarvs`i' - 1 if drugs_`j' != 0 & daysarvs`i' != .
            replace drugs_`i' = daysarvs`i' if drugs_`j' == 0 & daysarvs`i' != .
            replace drugs_`i' = drugs_`j' - 1 if daysarvs`i' == .
            replace drugs_`i' = 0 if drugs_`i' < 0
            replace drugs_has = (drugs_`i' != 0)
            replace days_without = days_without - drugs_has

}

*
*
*   Now drop some variables that are not needed before reshaping as long (panel data)


drop daysarvs* /*drugs_received_today*/ drugs_has days_without


reshape long drugs_ , i(ID) j(daysonarvs)
```

```
178
179 *  With a standard once a day, fixed-does triple combination pill, there is one pill per day.
180
181 * Save this panel data set as 3 -- Final panel dataset with pill counts after Part 3.dta
182
183
184
185
186
187
188
189 ********************************************************************************************************************************
    **********************
190 *
191 *
192 *                                                    PART 4
193 *
194 *
195 * PART 4  -- Now need to create additional time variables that link each daysonarvs to that date in relation to the date
    of delivery
196 *            and then create a full panel dataset
197 *
198 *
199 *
200 *
201 ********************************************************************************************************************************
    **********************
202
203
204 * First create a date variable that just shows the calendar date for exact daysonarvs variable in the data set
205
206 gen date = date_beginart + daysonarvs
207
208 format %td date
209
```

```
214 gen daystodelivery = date - date_delivery
215
216
217 *           daystodelivery is the time variable for estimating ARV coverage during differnt PMTCT periods
218 *           (e.g., -24 weeks to delivery, 1 years after delivery, etc.)
219
220 * And last, create a variable "hasarvs" on that day that is 0 if drugs_ == 0 and 1 if drugs_ > 0.
221
222
223 generate hasarvs = 0
224
225 replace hasarvs = 1 if drugs_ > 0
226
227
228
229
230 **** New -- Now create a full balance panel with daystodelivery as the time variable
231
232
233
234 tsset ID daystodelivery
235 tsfill, full
236 replace hasarvs = 0 if hasarvs == .
237
238 ** NOW FILL IN VARIABLES THAT ARE CONSTANT IN THE DATA SET
239
240
241 bysort ID (date_delivery) : replace date_delivery = date_delivery[_n-1] if missing(date_delivery)
242
243 bysort ID (date_ANC) : replace date_ANC = date_ANC[_n-1] if missing(date_ANC)
244
245 bysort ID (date_beginart) : replace date_beginart = date_beginart[_n-1] if missing(date_beginart)
246
247
248 * sort again as panel
```

```
252
253
254 * Now fill in missing dates due to tsset, full
255
256 replace date = date_delivery + daystodelivery if date == .
257
258
259 replace daysonarvs = date - date_beginart if daysonarvs == .
260
261
262 * optional -- delete observations before conception (days to delivery < -280) and beyond the evaluation period (example
    below is one year, so 365 days)
263
264 drop if daystodelivery < -280
265
266 drop if daystodelivery > 365
267
268
269 *           Save as 4 -- Final panel dataset after Part 4
270
271
272 ************************************************************************************************************************
    ************************
273 *
274 *
275 *
276 *                                                  Part 5 -- Collapse panel dataset over period of time for final outcome creation
277 *
278 *
279 *
280 *
281 ************************************************************************************************************************
    ************************
282
283
```

```
between -168 to day 0)
287 *       Create the final coverage variable (e.g., coverage_85p below meaning 85% of days were covered with ARVs between
    -168 and 0)
288
289
290
291
292
293
294 collapse (max) daysonarvs date_delivery date_ANC date_beginart (sum)hasarvs if daystodelivery >= -168 & daystodelivery <=
    0, by(ID)
295
296
297 * could collapse over other periods (e.g., final 8 weeks or pregnancy, first 24 weeks post partum, and so on).
298
299
300 label variable daysonarvs "(max) daysonarvs (days from initiation to delivery)"
301
302
303 * Note:  In this example, in the collapsed dataset, "daysonarvs" is how many days the women was on ARVs at the time of
    delivery.
304 *  For example, daysonarvs = 90 means the women initiated ART 90 days before delivery; daysonarvs = -90 means the women
305 *  initiated 90 days after delivery.
306
307
308 label variable hasarvs "(sum) hasarvs (number of days with ARVs during collapsing period)"
309
310
311 gen coverage_24 = hasarvs/168
312
313
314 * Note: if alternative period (such as final 8 weeks of pregnancy, then need to chance 168 in previously line to
    appropriate number).
315
316
```

```
324
325
326  * Save collapsed data set as 5 -- Final outcome 24 weeks to delivery
327
328
329  * DONE
330
331
332
333
334
```

334
